# *Ureaplasma* and *Prevotella* colonization with *Lactobacillus* abundance during pregnancy facilitates term birth

**DOI:** 10.1038/s41598-022-13871-1

**Published:** 2022-06-16

**Authors:** Sunwha Park, Young-Ah You, Young-Han Kim, Eunjin Kwon, AbuZar Ansari, Soo Min Kim, Gain Lee, Young Min Hur, Yun Ji Jung, Kwangmin Kim, Young Ju Kim

**Affiliations:** 1 Department of Obstetrics and Gynecology, College of Medicine, Ewha Medical Research Institute, Ewha Womans University, 1071, Anyangcheon-ro, Yangcheon-gu, Seoul, 07985 Korea; 2 Department of Obstetrics and Gynecology, College of Medicine, Yonsei University, Seoul, Korea; 3 3BIGS CO., LTD., Seoul, Korea

**Keywords:** Translational research, Reproductive disorders, Microbiology, Medical research, Risk factors

## Abstract

*Ureaplasma* and *Prevotella* infections are well-known bacteria associated with preterm birth. However, with the development of metagenome sequencing techniques, it has been found that not all *Ureaplasma* and *Prevotella* colonizations cause preterm birth. The purpose of this study was to determine the association between *Ureaplasma* and *Prevotella* colonization with the induction of preterm birth even in the presence of *Lactobacillus*. In this matched case–control study, a total of 203 pregnant Korean women were selected and their cervicovaginal fluid samples were collected during mid-pregnancy. The microbiome profiles of the cervicovaginal fluid were analyzed using 16S rRNA gene amplification. Sequencing data were processed using QIIME1.9.1. Statistical analyses were performed using R software, and microbiome analysis was performed using the MicrobiomeAnalyst and Calypso software. A positive correlation between *Ureaplasma* and other genera was highly related to preterm birth, but interestingly, there was a negative correlation with *Lactobacillus* and term birth, with the same pattern observed with *Prevotella. Ureaplasma* and *Prevotella* colonization with *Lactobacillus* abundance during pregnancy facilitates term birth, although *Ureaplasma* and *Prevotella* are associated with preterm birth. Balanced colonization between *Lactobacillus* and *Ureaplasma* and *Prevotella* is important to prevent preterm birth.

## Introduction

Preterm birth (PTB) is characterized as the delivery of newborns at less than 37 weeks of gestation and is a major cause of morbidity and mortality among infants^1^. In preterm infants, respiratory and cardiovascular complications are generally observed, which can result in neonatal death, as well as long-term complications, such as neurodevelopmental delay, hearing and visual loss, and cerebral palsy, leading to significant socioeconomic loss^2^. The risk factors for PTB are influenced by various causes such as ethnicity, education level, smoking, obesity, environmental effects, and underlying diseases^3^. The recent increase in PTB is affected by rising in the number of elderly and multiple pregnancies. This has prompted an increase in research to diagnose and prevent PTB^4,5^.

Spontaneous PTB (sPTB) accounts for 70–75% of all PTBs^1,6^. One-third of sPTBs are accompanied by intra-amniotic infections, and the isolated microorganisms, especially *Ureaplasma*, are similar to those observed in the lower genital tract^7^. Hence, infection/inflammatory response attributed to ascending infections was thought to be the cause^8^. Increased levels of inflammatory cytokines in the amniotic fluid, cervicovaginal fluid (CVF), and blood support this hypothesis^9–11^. Ascending infections contributing to sPTB are related to the vaginal environment, and this risk is high in bacterial vaginosis (BV), which involves the proliferation of harmful bacteria^12^. BV can be identified using a culture test, by microscopy, or measurement of vaginal pH. With the development of molecular genetic diagnostic techniques, including 16s rRNA metagenomics analysis, it became possible to detect various bacterial species, including anaerobic bacteria, and to obtain genetic information of all bacterial species distributed in bio-fluid^13^.

In pregnant women, *Lactobacillus* becomes dominant in the vaginal environment because of marked increases in circulating estrogen^14^. Specifically, the *Lactobacillus* group tends to be dominant in women with TB^15^. According to the dominant *Lactobacillus spp*., there have also been studies that reported on the relationship with PTB by classifying them into community state types (CST)^16,17^. In contrast, microbiome dysbiosis, a state of imbalance in the microbial community, particularly in the vaginal microbiome, is related to PTB^18^. Among various bacteria, several studies have reported on the relationship between *Ureaplasma* and *Prevotella* and PTB. In addition, the effect of the microbiome of pregnant women on the prognosis during pregnancy and PTB has been studied^19–23^.

However, according to previous studies, the vaginal microbiome shows differences according to race and place of residence^18,24^, and a study has also reported that there is no relationship between PTB and a specific microbiome in some races^17^. *Ureaplasma* spp. is known to be associated with PTB, however, vaginal colonization does not universally result in induction of ascending infection and pregnancy complications, even for animal models^25,26^.

Therefore, in this study, we aimed to evaluate candidates that can be a predictor of PTB among the microbiome of CVF and to determine the association between *Ureaplasma* and *Prevotella* colonization with PTB and whether it induces PTB even in the presence of *Lactobacillus*.

## Results

### Clinical characteristics

**Figure 1:**
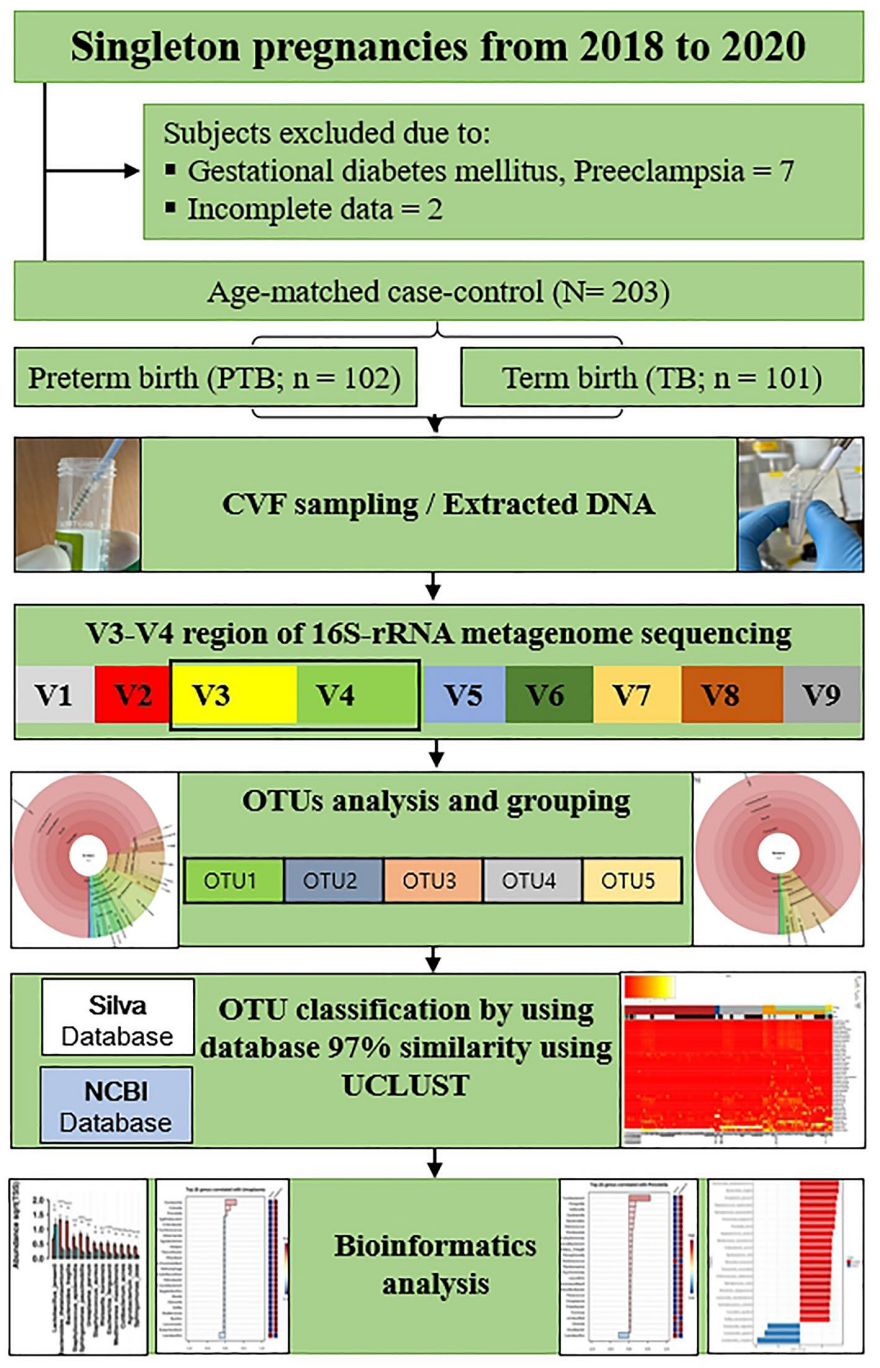
Flowchart of the study. *CVF* cervicovaginal fluid, *rRNA* ribosomal ribonucleic acid, *OTUs* operational taxonomic units.

A total of 203 women participated in this age-matched case–control study, excluding nine subjects: 102 women in the PTB group and 101 women in the TB group (Fig. 1). There were no significant differences between the characteristics of the PTB and TB groups, except for the history of sPTB, white blood cell (WBC) count, cervical length (CL), gestational age at sampling (GAS), gestational age at birth (GAB), birth weight, appearance, pulse, grimace, activity, respiration (APGAR) score, and neonatal intensive care unit (NICU) admission rate (*P* < 0.001, Table 1).

**Table 1:** Clinical characteristics of the study subjects.

Characteristics	Preterm birth (n = 102)	Term birth (n = 101)	P-value
Maternal age (year)	32.32 (± 4.35)	33.02 (± 3.40)	0.237
Pre-pregnancy BMI (kg/m^2^)	21.60 (± 2.89)	21.36 (± 2.77)	0.657
**Education level**			> 0.999
High school graduation or below	6 (13.3)	11 (15.3)	
University graduates	39 (86.7)	61 (84.7)	
**History of PTB**			< 0.015*
No	79 (88.8)	94 (97.9)	
Yes	10 (11.2)	2 (2.1)	
**WBC**	10.1 (8.8–12.9)	8.9 (7.8–10.4)	< 0.001*
Cervical length	20.8 (± 12.1)	30.4 (± 8.5)	< 0.001*
GAS (weeks)	31.8 (26.2–34.2)	24.5 (22.1–31.0)	< 0.001*
**CST**			0.156
I	46 (45.1)	45 (44.6)	
II	1 (1.0)	4 (4.0)	
III	20 (19.6)	22 (21.8)	
IV-A	7 (6.9)	1 (1.0)	
IV-B	23 (22.5)	27 (26.7)	
V	5 (4.9)	2 (2.0)	
**Delivery mode**			0.004*
ND	41 (40.2)	61 (60.4)	
CS	61 (59.8)	40 (39.6)	
GAB (weeks)	34.0 (30.4–35.6)	39.3 (38.2–39.9)	< 0.001*
Birth weight (g)	1975.4 (± 780.5)	3234.9 (± 316.8)	< 0.001*
APGAR score at 1 min	6.99 (5–9)	9.31 (9–10)	< 0.001*
APGAR score at 5 min	8.29 (7–10)	9.74 (10–10)	< 0.001*
NICU admission	86 (84.3)	13 (12.9)	< 0.001*

Categorical variables were expressed as frequencies (percentage) and analyzed using the chi-square test and Fisher’s exact test. Continuous variables were expressed as the mean ± standard deviation (SD) or median (interquartile range) and were compared using the *t*-test or Mann–Whitney U test.

*BMI* body mass index, *PTB* preterm birth, *WBC* white blood cell, *GAS* gestational age at sampling, *CST* community-state type, *ND* normal delivery, *CS* cesarean section, *GAB* gestational age at birth, *APGAR* appearance, pulse, grimace, activity, respiration, *NICU* neonatal intensive care unit.

*Statistical significance was defined as P < 0.05.

### Association between bacteria and preterm birth

#### Differences in microbial diversity between PTB and TB groups

**Figure 2:**
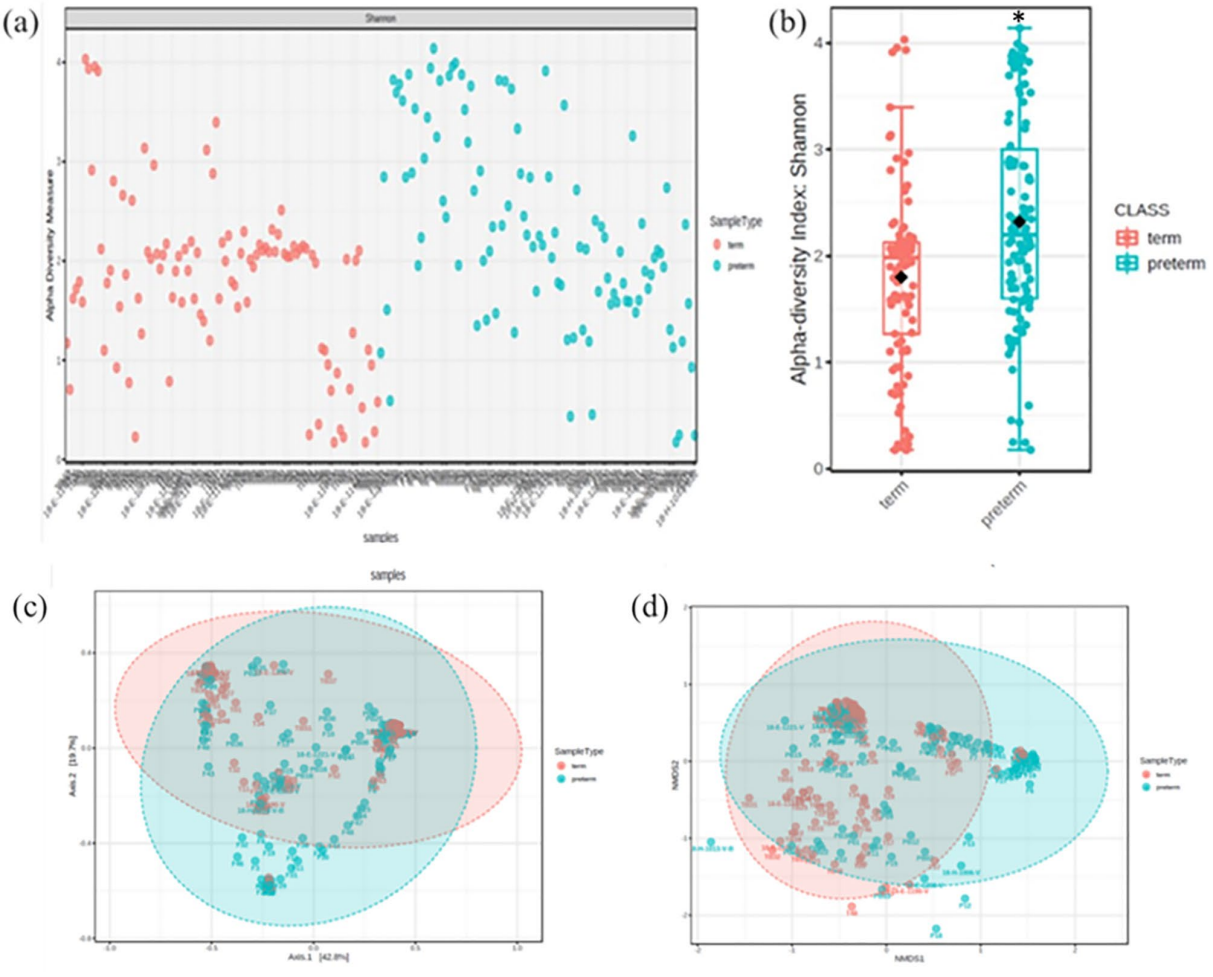
Differences in alpha- and beta-diversity between PTB and TB groups. (**a**,**b**) Shannon's alpha diversity was significantly higher in the PTB group (PTB, n = 102; TB, n = 101). (**c**) Principal coordinate analysis plot (**d**) Non-metric multidimensional scaling plot. Boxes show median and interquartile ranges, black dots represent the average, and whiskers extend from minimum to maximum values. The asterisk symbol shows a significant difference. *Statistical significance was defined as *P* < 0.05.

Comparing the Krona chart with PTB and TB, PTB showed that *Bacteroidetes, Proteobacteria*, and *Mollicutes* were diversely distributed from the phylum level in addition to *Firmicutes* and *Actinobacteria* (Supplementary Fig. 1). In the TB group, 88% consisted of *Lactobacillus*, and *Gardnerella, Bifidobacterium*, and *Atopobium* accounted for a small percentage. In the PTB group, *Lactobacillus* accounted for 69% and the distribution of other species such as *Staphylococcus, Bacteroides, Prevotella, Ureaplasma, Sphingomonas,* and *Escherichia* was shown (Supplementary Fig. 1). When comparing Shannon’s α-diversity index between the PTB and TB groups, the medians (interquartile ranges) were 2.20 (1.27–2.12) and 1.99 (1.60–3.03), respectively. The median was significantly higher in the PTB group (*P* < 0.001, Fig. 2a,b). In the β-diversity analysis, most of the microbial communities were similar, but a distance was observed between specific microorganisms (Fig. 2c,d). There was no significant difference in the CST type between the PTB and TB groups (*P* = 0.156, Table 1 and Supplementary Fig. 2).

#### Microbiome related to the PTB and TB groups

A total of 157 species were detected, of which 82 species had an adjusted *P *value of < 0.05, and the median values were compared for the top 20 bacteria (Supplementary Fig. 3). In the analysis of the two groups, the abundances of *Lactobacillus jensenii*,* Bacteroides thetaiotaomicron*,* B. fragilis*,* Staphylococcus epidermidis*,* Sphingomonas paucimobilis*,* Ureaplasma parvum*,* S. aureus*,* Weissella koreensis*,* Escherichia fergusonii*,* Mediterranea massiliensis*,* Cutibacterium acnes*,* Agrobacterium rubi*, and *S. zeae* were significantly different (Supplementary Fig. 4, *P* < 0.05, abundance > 0.5%).

**Figure 3:**
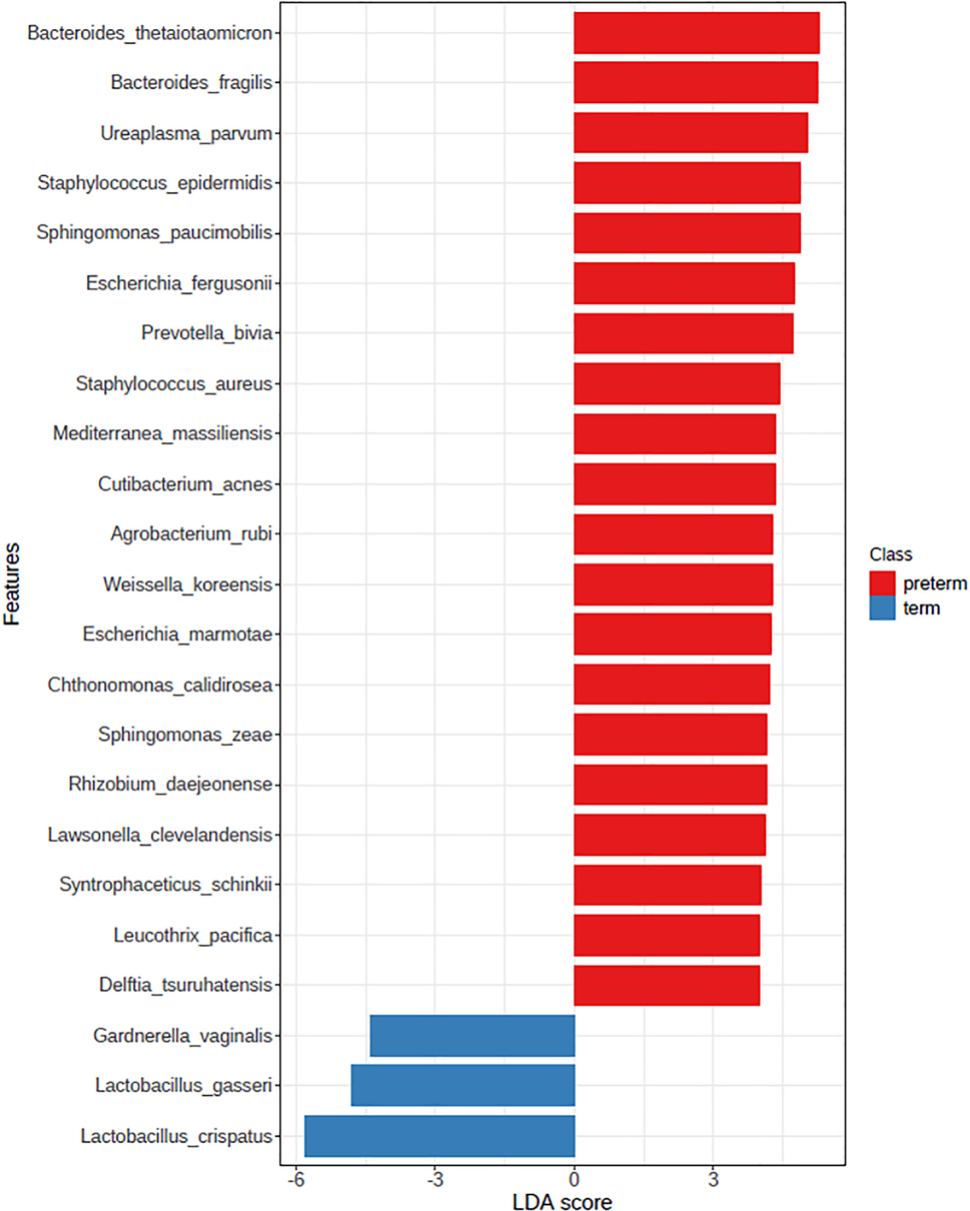
Differential dominant relative abundance of bacterial taxa in the PTB and TB groups. Linear discriminant analysis effect size analysis (LDA score > 4). *LDA* Linear discriminant analysis.

A total of 23 significant species were observed, with 20 in the PTB group and three in the TB group (*P* < 0.05, LDA score of ≥ 4). *L. crispatus*,* L. gasseri*, and* Gardnerella vaginalis* were the major taxa in the TB group*. B. thetaiotaomicrom*,* B. fragilis*,* U. parvum*,* S. epidermidis*,* S. paucimobilis*,* E. fergusonii*,* Prevotella bivia*,* S. aureus*,* M. massiliensis*,* C. acnes*,* A. rubi*,* W. koreensis*,* E. marmotae*,* Chthonomonas calidirosea*,* S. zeae*,* Rhizobium daejeonense*,* Lawsonella clevelandensis*,* Syntrophaceticus schinkii*,* Leucothrix pacifica*, and* Delftia tsuruhatensis* were the major taxa in the PTB group (Fig. 3).

#### Pattern search using correlation analysis of the vaginal microbiome

**Figure 4:**
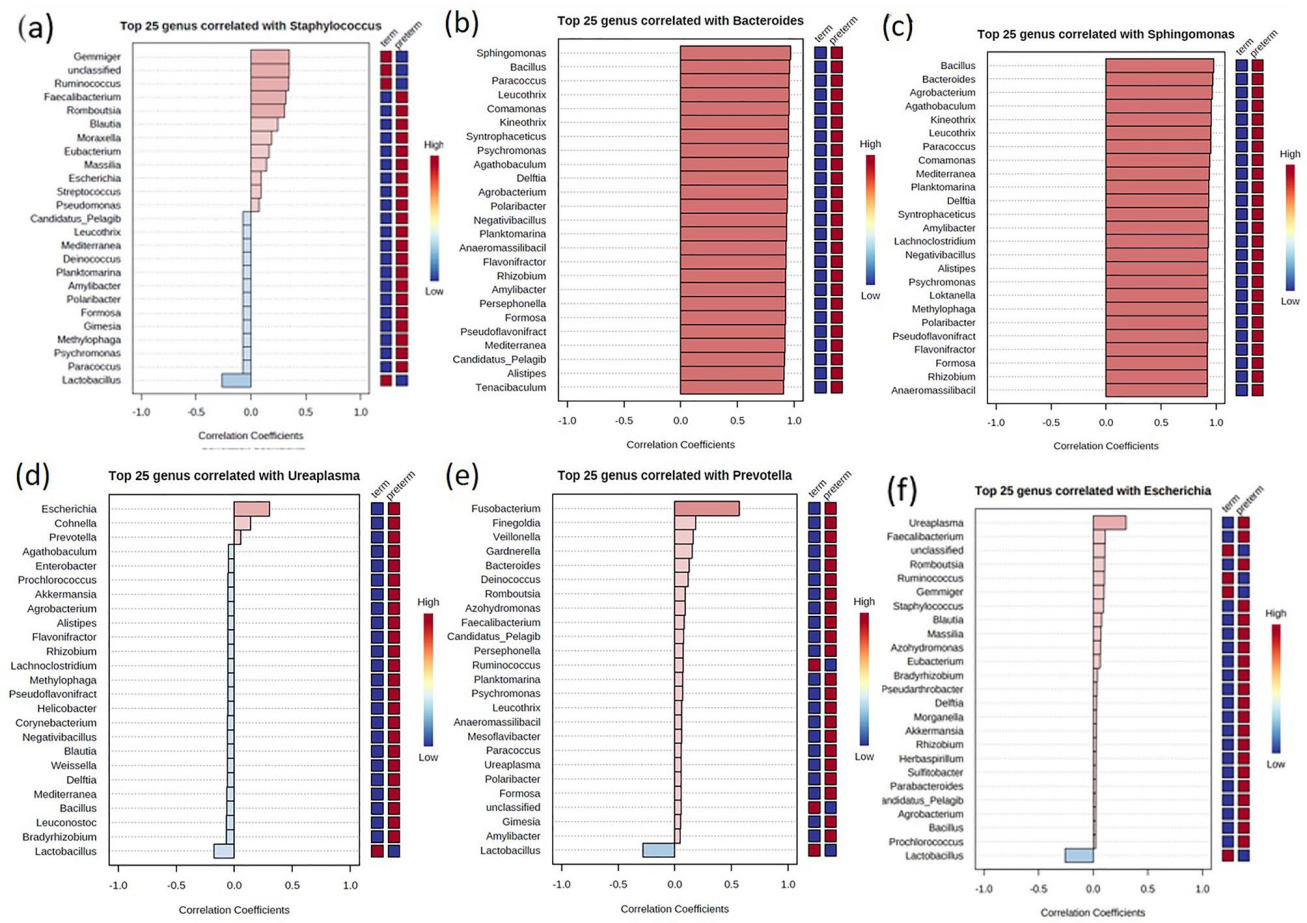
Pattern search using correlation coefficient analysis of the vaginal microbiome of pregnant women at the genus level. (**a**) *Staphylococcus* (**b**) *Bacteroides* (**c**) *Sphingomonas* (**d**) *Ureaplasma* (**e**) *Prevotella* (**f**) *Escherichia.*

When the examination was performed at the representative genus level to identify significant differences in pattern using correlation analysis, *Staphylococcus* showed a positive correlation of 0.3 with *Gemmiger* and *Ruminococcus* and showed a negative correlation of 0.3 with *Lactobacillus,* and in this case, the association with TB was high (Fig. 4a). *Bacteroides* showed a positive correlation of ≥ 0.8 with 43 genera and a negative correlation of 0.6 with *Lactobacillus* (Fig. 4b). *Sphingomonas* showed a positive correlation of ≥ 0.8 with 46 genera and a negative correlation of 0.6 with *Lactobacillus* (Fig. 4c). *Ureaplasma* had a correlation of 0.3 with *Escherichia*, and *Prevotella* had a positive correlation of 0.5 with *Fusobacterium* (Fig. 4d,e). When *Ureaplasma* showed a negative correlation with *Lactobacillus*, the subjects associated with TB were marked in red on the right box line (Fig. 4d). Similarly, in the case of *Prevotella*, negative correlations with *Lactobacillus* were highly correlated with TB. In addition, positive correlations with *Ruminococcus* were also associated with TB (Fig. 4e). Escherichia showed a positive correlation with *Gemmiger* and *Ruminococcus* and a negative correlation with *Lactobacillus*, and in this case, the association with TB was high (Fig. 4f).

## Discussion

This is the first study to conduct correlation analysis with bacteria of the vaginal microbiome, and we suggest that the relevance to the PTB should be evaluated by the community, not just the presence of specific bacteria. Although *Ureaplasma* and *Prevotella* are related to PTB, if these bacteria coexist with *Lactobacillus*, pregnancy outcome will be followed by TB.

These results were obtained by comparing the CVF microbiome of the PTB and TB groups using 16s rRNA metagenome sequencing. Through this method, we were able to understand the entire ecosystem of the vagina and identified 20 candidate bacteria related to PTB and three bacteria related to TB among all colonizing microbiomes. The *Lactobacillus* spp. and *Gardnerella vaginalis* were dominant in the TB group, whereas *Bacterioides* spp*., Ureaplasma* spp.,* Staphylococcus* spp.,* Escherichia fergusonii*,* Prevotella* spp., and *Sphingomonas* spp. etc. were increased in the PTB group. However, although bacteria related to PTB were present, there were cases where delivery became TB, which coexisted with *Lactobacillus*, *Ruminococcus*, and *Gemmiger*.

Our study results were similar to those of previous studies that used 16s rRNA metagenome sequencing. When comparing between the PTB with TB groups, *Lactobacillus* spp., including *L. crispartus*,* L. jensenii,* and *L. gasseri*, were associated with TB^18^. In pregnant women, the vaginal microbiome is dominated by *Lactobacillus* from the second trimester, reducing bacterial diversity^27^. Vaginal *Lactobacillus* spp. is known to protect the vaginal environment from harmful bacteria as beneficial bacteria, which is thought to be because lactic acid or hydrogen peroxide secreted by *Lactobacillus* plays a bacteriostatic role. Therefore, in the group with a lower dominance of *Lactobacillus*, the risk of PTB increases with the result of increasing pathogens^15,19,28,29^.

In contrast to the findings of previous studies^30,31^, we found that *G. vaginalis* was associated with TB. However, controversy exists because there were positive findings in healthy women^31^. Thus, we realized that *G. vaginalis* needed to be identified up to the strain level^32^.

Among the increased bacteria in PTB cases, *B. fragilis, U. parvum*, and *P. bivia* have been associated with PTB in previous studies^23,33,34^. *Staphylococcus* spp., *Escherichia* spp., *and Sphingomonas* spp. are commonly known as opportunistic bacteria^35–37^. In our study, *B. thetaiotaomicron* and *B. fragilis* were found to increase in PTB cases. *Prevotella* spp. is one of the genera known to be associated with PTB and BV^33^. The *Mollicutes* class, which includes *Ureaplasma* and *Mycoplasma*, has also been studied for its potential association with PTB^22,34^, and this is also associated with the cause of a shorter cervical length^38^. However, there was controversy about colonization in healthy women, and this study revealed that colonization itself was not associated with all infection and pregnancy complications^21^.

Therefore, our research is meaningful because, in the analysis of the vaginal microbiome, we revealed the importance of interpretation through an understanding of the microbiome relationship. *Ureaplasma* and *Prevotella*, previously considered pathogenic, did not cause complications when coexisting with *Lactobacillus* in our study. Furthermore, while a previous study showed cervical epithelial damage increased PTB, the protective role of *Lactobacillus* may explain why experimental *Ureaplasma* infection only resulted in a maximum of 28% induced PTB^26^. This was consistent with the bacterial risk score model, a study that analyzed the microbiome using machine learning, developed by this research team to predict PTB^21^. In this study, *Ruminococcus* and *Gemmiger* were suggested to play a protective role against pathogenic bacteria. They have been studied as part of a healthy gut microbiome, so we suggest the possibility of an association with the healthy intestinal microbiome and TB^39,40^.

The results of this study using correlation analysis suggest that *Lactobacillus* is thought to show a protective effect against *Ureaplasma* and *Prevotella* as well as most pathogenic bacteria. However, in this study, only the results using bioinformatics were shown, and no experiments were conducted to reveal the causal relationship. Therefore, for future research, in order to confirm the biological mechanism, it may be necessary to study the analysis of proteomics and metabolomics in addition to genomics. Furthermore, studies such as changes in cytokines or immune activation to determine how this microbiome acts with the host should be conducted together.

To our knowledge, this is the first study to examine the microbiome relationship using pattern search through correlation analysis. Through this study, it was suggested that the relationship through the quantitative analysis of the microbiome, not simply the presence or absence of pathogenic bacteria, would be more important in predicting PTB. Furthermore, after target selection through metagenome analysis, multiplex quantitative PCR (qPCR), which can save relatively cost and time, can be used in clinical practice for predicting PTB^21^. This study has strengths as a large-scale, multicenter study targeting pregnant Korean women. The limitations of this study are that it was not analyzed, including strain level measurements for *U. parvum* and *G. vaginalis*, despite recent studies showing that the pathogenicity of *Ureaplasma* and *Gardnerella* differs depending on the serovar or clade levels. In addition, this study may be limited in that it did not analyze the microbiome in amniotic fluid or placenta to confirm intra-amniotic infection. As a limitation of the method itself, 16s rRNA metagenome sequencing can analyze all colonized microbiome of the vagina with high sensitivity, but it is difficult to identify the actual activity and pathogenicity of the microbiome. However, despite these limitations, candidates related to PTB were discovered through various bioinformatics analyses to understand their relationship.

*Ureaplasma* and *Prevotella* colonization with *Lactobacillus* abundance during pregnancy facilitates TB, although *Ureaplasma* and *Prevotella* are associated with PTB. Balanced colonization between *Lactobacillus* and *Ureaplasma* and *Prevotella* is important for preventing PTB.

## Methods

### Study subjects and CVF collection

In this case-cohort study, subjects were recruited from the Ewha Womans University Mokdong Hospital and Yonsei University Severance Hospital from 2018 to 2020. This study was approved by the Ethical Research Committee of Ewha Womans University Mokdong Hospital (no. 2018-07-007) and Yonsei University Severance Hospital (no. 4-2018-0564). The experiments were conducted in accordance with the approved guidelines, and informed consent was obtained from all the subjects.

Subjects included women with a singleton pregnancy and at a gestational age between 15 and 36 weeks, asymptomatic pregnant women who visited the outpatient clinic department regularly, and hospitalized pregnant women with symptoms of PTL or preterm premature rupture of membranes. The CVF sample was collected from the posterior vaginal fornix using sterile cotton swab through the speculum exam before any vaginal examination or clinical treatment, such as antibiotics, steroids, progesterone, and tocolytics.

For all study subjects, baseline demographic data and health-related characteristics including age, pre-pregnancy body mass index, education level, and maternal PTB history were collected. A routine blood test was performed to examine the WBC CL at the time of CVF collection. After delivery, the outcomes of the pregnancy were evaluated, including delivery mode; GAB; neonatal birth weight; APGAR score; and NICU admission. Among the enrolled subjects, those diagnosed with gestational diabetes mellitus, preeclampsia, and insufficient medical records were excluded. The PTB group was categorized as subjects who delivered at < 37 weeks of gestation, whereas the TB group was characterized by subjects who delivered after 37 weeks of gestation.

### Metagenome analysis using 16s rRNA gene sequencing

The collected samples were subjected to bacterial DNA extraction for microbiome analysis using the NucleoSpin Tissue Kit (Macherey-Nagel, Düren, Germany) following the manufacturer's instructions. A 16S rRNA sequencing library was constructed according to the 16S metagenomic sequencing library preparation protocol targeting the V3 and V4 hypervariable regions of the 16S rRNA gene^41–43^. The KAPA HiFi HotStart ReadyMix (KAPA Biosystems, Wilmington, USA) and Agencourt AMPure XP system (Beckman Coulter Genomics, Brea, USA) were used for PCR and purification of the PCR product, respectively. The initial PCR was performed with 12 ng of the template DNA using region-specific primers that were compatible with the Illumina index and sequencing adapters (Supplementary Table 1). After magnetic bead-based purification of PCR products, a second PCR was performed using primers from the Nextera XT Index Kit (Illumina, San Diego, USA) with a limited cycle. Subsequently, purified PCR products were visualized using gel electrophoresis and quantified with a DropSense96 (Trinean, Gentbrugge, Belgium). The pooled samples were run on the Agilent 2100 Bioanalyzer (Agilent, Santa Clara, CA, USA) for quality analysis prior to sequencing. Libraries were quantified by qPCR using the CFX96 Real-Time System. After normalization, sequencing of the prepared library was conducted using the MiSeq system (Illumina, San Diego, USA) with 300 bp paired-end reads.

Sequencing data were processed using QIIME1.9.1 to assemble paired-end reads into tags according to their overlapping relationships. In the pre-processing step, the primer was removed, demultiplexed, and filtered for quality (Phred ≥ 20). USEARCH7 was used to perform denoising and chimera detection/filtering in the operational taxonomic unit (OTU) group. Then, the Silva132 and NCBI databases were used to determine the OTUs with 97% similarity using UCLUST and the close-reference analysis method and to determine the OTU identifiers. Comparative OTU assignment was performed with the database in terms of phylum, class, order, family, genus, and species separately using RDP classifiers. Using QIIME, the α-diversity was analyzed with the Shannon index to understand the local population of the microbiome and the β-diversity was analyzed for estimating the correlation among other factors and microbes by Bray–Curtis.

Basic statistical analyses, such as *t*-test, Mann–Whitney U test, Chi-square test, Fisher’s exact test, heat map, Krona chart, and the linear discriminant analysis (LDA) effect size (LEfSe) were performed. Multivariate analyses, such as principal coordinate analysis and non-metric multidimensional scaling, were performed. The adjusted *P* value was calculated by adjusting the false-positive rate using the false discovery rate. Correlations between the taxa and sample groups were analyzed using the Pearson correlation coefficient r as the distance measure. Statistical analyses were performed using R software (version 3.6.2), and microbiome analysis was performed using the MicrobiomeAnalyst (https://www.microbiomeanalyst.ca/) and the Calypso (http://cgenome.net/Calypso/) software.

## Supplementary Information

Supplementary Information.
