# Supplementary material for: Ureaplasma and Prevotella colonization with Lactobacillus abundance during pregnancy facilitates term birth
Source: Sci Rep. 2022 Jun 16;12:10148. doi: 10.1038/s41598-022-13871-1 (PMC9203766; doi:10.1038/s41598-022-13871-1)
Supplement: Supplementary file 1 — Supplementary Information. [file 41598_2022_13871_MOESM1_ESM.docx]

**Supplementary**

**Supplementary Table 1.** Region-specific primers

| Forward primer | 5′-TCGTCGGCAGCGTCAGATGTGTATAAGAGACAGTCGTCGGCAGCGTCAGATGT  GTATAAGAGACAGCCTACGGGNGGCWGCAG-3′ |
| --- | --- |
| Reverse primer | 5′-GTCTCGTGGGCTCGGAGATGTGTATAAGAGACAGGTCTCGTGGGCTCGGAGA  TGTGTATAAGAGACAGGACTACHVGGGTATCTAATCC-3′ |


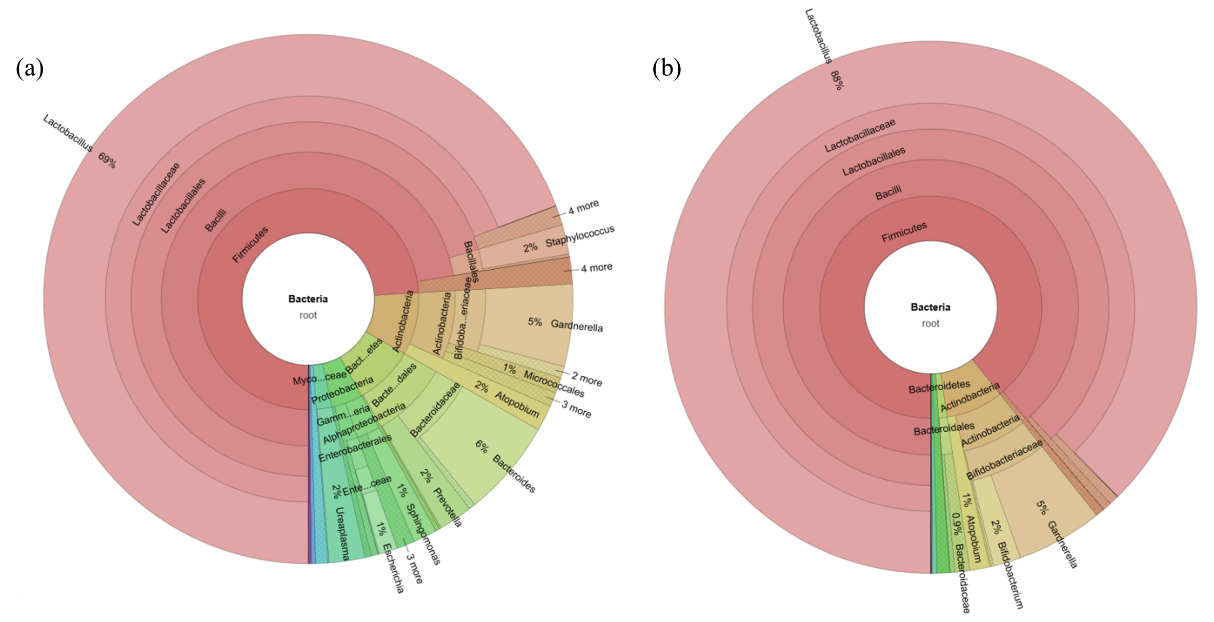

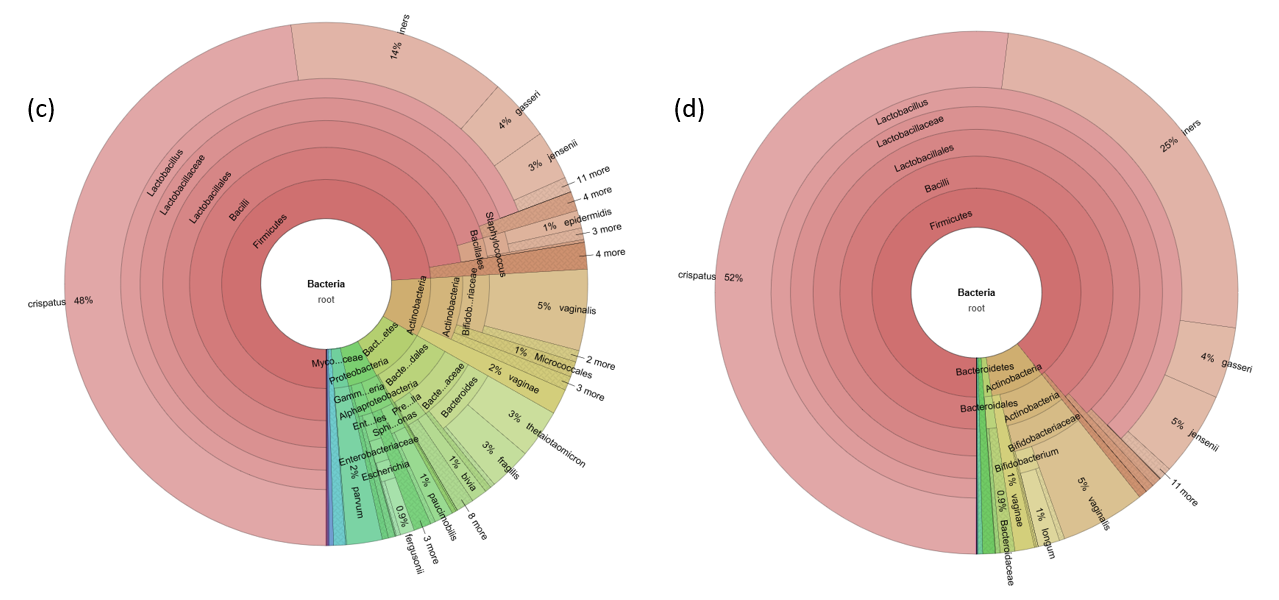


**Supplementary Fig 1.** Krona chart of bacterial taxa between the preterm birth and term birth groups.

(a), (c) show the preterm birth group (n=102) with genus level and species level. (b), (d) show the term birth group (n=101) with genus level and species level

**
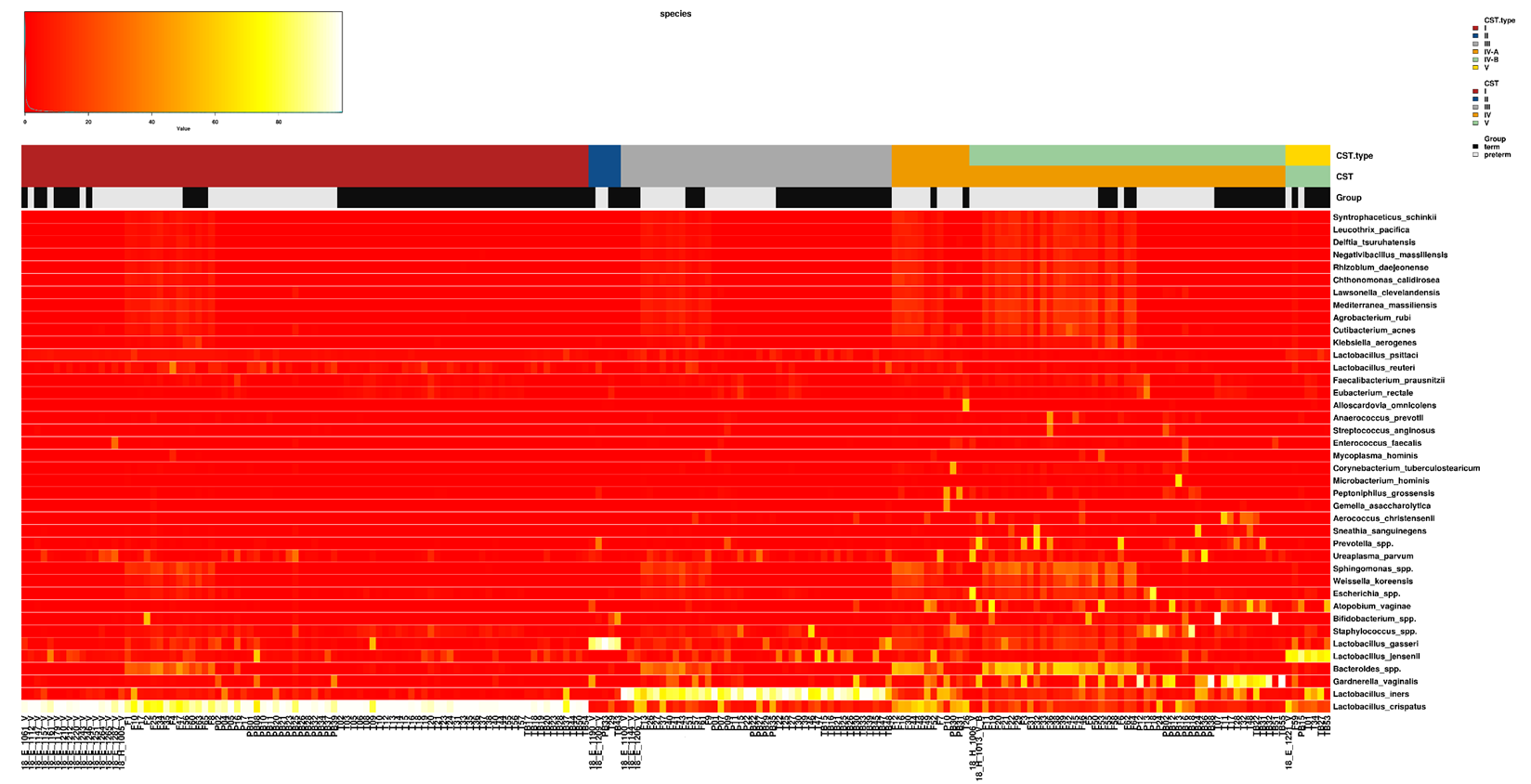
**

**Supplementary Fig 2.** Heatmap of microbial taxa of 203 subjects according to the CST type

CST, community-state types; CST-I, *Lactobacillus crispatus*; CST-II, *Lactobacillus gasseri*; CST-III, *Lactobacillus iners*; CST-IV-A, *Heterogeneous type, other Lactobacillus* spp.; CST-IV-B, *fewer lactobacilli and more anaerobic bacterial taxa*; CST-V, *Lactobacillus jensenii*


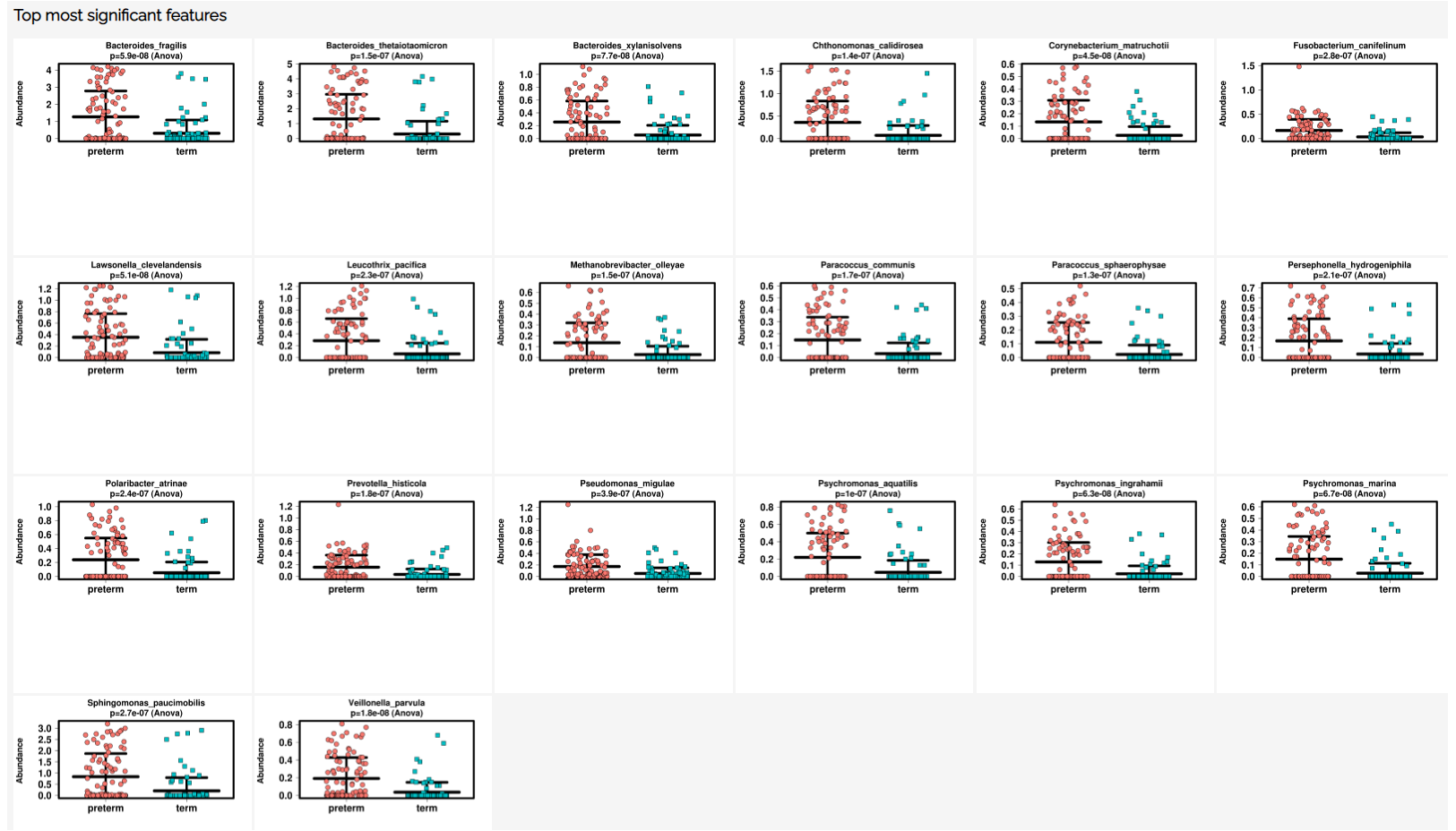


**Supplementary Fig 3.** Top 20 bacterial communities showing significant features related to preterm birth

The 16s rRNA abundance of the taxa differed significantly between the preterm birth (n=102) and term birth (n=101) groups. Statistical significance was observed using the Mann-Whitney U test, and the adjusted *P*-value was calculated by adjusting the false positive rate using the false discovery rate.

Whiskers show the median and the interquartile range.


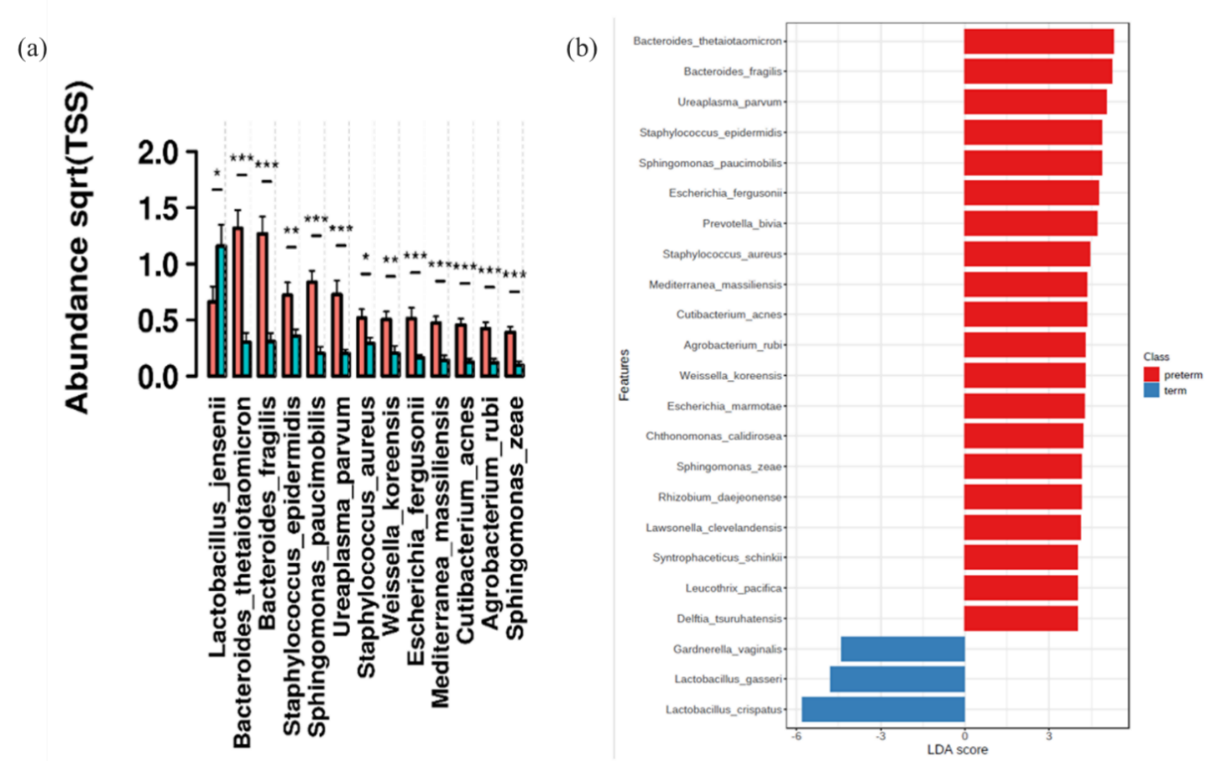

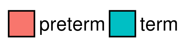


**Supplementary Figure 4.** Differential dominant relative abundance of bacterial taxa in the PTB and TB groups.

Thirteen bacteria showing significant differential mean relative abundance determined using Mann-Whitney U test (abundance >0.5 %) *Significance between groups (*: *P*< 0.05 / **: *P* < 0.02 / ***: *P* < 0.005),

sqrt, square-root transformed; TSS, Total Sum Scaling.
